# On a New Mode of Treatment Employed in the Cure of Various Forms of Ulcer and Granulating Wounds

**Published:** 1838-01

**Authors:** 


### Art. XVII.

*On a new Mode of Treatment employed in the Cure of various Forms of Ulcer and granulating Wounds.* By F. C. Skey, F.R.S., Assistant Surgeon to St. Bartholomew's Hospital.—*London*, 1837. 8vo. pp. 85.

As its title leads us to expect, this treatise is almost exclusively practical. Whatever temptation the subject of the pathology of ulceration may have offered to its author, he has almost entirely resisted; with the exception of some few remarks which connect themselves with the supposed *modus operandi* of the treatment recommended. The work is divided into four parts: the first, containing some general remarks on ulceration and ulcers; the second, on some of the medical properties of opium, the remedy proposed for them; the third, on the general treatment of ulcers and cicatrizing wounds; and the fourth, cases illustrative of the effects of the treatment proposed. We shall shortly analyze each part. Practical men (and the book is not intended for others,) know what is meant by an ulcer; so that we need not puzzle them with definitions. The classification adopted by Mr. Skey is that of Sir E. Home, and is a very useful one,—viz. ulcers characterized by excess, and those characterized by torpidity of action. The processes which constitute the healing of an ulcer,—exudation of lymph, its organization and cicatrization,—are dependent for their activity on the activity of the general circulation, supposing them to be uninfluenced by art; for, of course, local action may be excited by local means, without the participation of the general system. As a general rule, subject, however, to important exceptions, an ulcer may be regarded as a good criterion of the vital energies; these energies diminishing proportionally according to the distance from the trunk. Mr. Skey broaches the old question, why ulcers on the legs are proportionally so much more frequent than in other parts? and he agrees with no explanation of this fact which has hitherto been offered. His reasons, however, against the usual explanation of what are termed varicose ulcers are very unsatisfactory; because "ulcers frequently exist without varix, and varix without ulcer, and both these forms of disease occur more frequently alone than do the two conjointly," is, supposing the statement to be a fact, no reason why varix may not be one of the frequent causes of ulcers. Might it not with as much justice be said, we have frequently extravasations of blood without blows, and blows without extravasations of blood, and both circumstances occur more frequently alone than do the two conjointly; therefore, blows cannot cause extravasations of blood. Both the blow and the varix (or the conditions essentially connected with the varix,) are mechanical causes, or are assumed to be such in the theory of their action; and, in one case, the extravasation, in the other the impeded circulation, is supposed to be thus mechanically produced. But Mr. Skey cannot see how varicose veins can act as any impediment to the arterial capillaries which transmit the blood to them. "If," he says, "the size of the vena saphena were diminished instead of increased, I could understand the cause of obstruction in the small veins supplying it; and I should suppose, except for the contrary authority of Sir E. Home, that, the larger the trunk of a vein, the greater the facility afforded for the transmission of blood into it from the smaller branches; and that, instead of obstruction of the capillaries, there would be every encouragement afforded to the arterial blood to advance." Can Mr. Skey, when he considers the anatomical structure of varicose veins, and the laws which govern the circulation, as far as we are cognizant of them, still maintain that an enlarged vein, probably deprived of its valves, or possessing them in such a condition as renders them incapable of performing their functions, affords greater facility for the transmission of blood into it? As reasonably might he talk of the increased contractility of an over-distended bladder. The effect of pressure in mitigating the evil but adds evidence to the great probability of the theory usually applied to the explanation of varicose ulcers.

We should not have devoted so much space to the consideration of this subject, but, when well-received and apparently rational explanations of morbid phenomena are attacked, the reasons for so doing should be satisfactory, and, if possible, the substitute should be equally so. Mr. Skey refers the cause farther back in the circulation,—i. e. "to the capillary system, *which wants power for transmission;*" and he goes on to say, that "it appears to me that nearly all the phenomena of disease in those parts of the body remote from the heart, denoting this languid action, may be referred to this system. The fault is not with the veins, but with the arteries, and this transmission is by them effected so languidly as in a great degree to deprive the smaller veins of the influence of their contractility. Thus, the blood accumulates in the larger venous trunks, which become knotted and convoluted to a degree quite surprising." We give Mr. Skey's hypothesis in his own words. No very potent argument is adduced for its truth; but, as we are not the advocates of any one exclusive mode by which varix may be produced, or on which the various processes of ulcer depend, we can imagine that, in certain cases, the fault may originate with the arterial capillaries, whilst in others we can conceive a similar state of these vessels as the result of some mechanical obstacle, such as a varicose vein, &c.

To this condition of the arterial capillaries, the author, and with plausibility, ascribes the weak granulations of distant parts of the body. Inflammation, which is essential to ulceration, is not essential to constitute an ulcer; so that depletion, which may be of service in the former case, is only so in the latter when, from causes foreign to the disease itself, some degree of inflammation has been superadded. The reproductive power of ulcer is quickly exhausted, if allowed to fall below the standard of health. The form presented by the ulcers "too weak to carry on the actions necessary for their recovery," is that of large, whitish, bloodless, insensible granulations: the smaller the granulations, the greater is their manifestation of health. In the former, there is need of artificial excitement. In one form of irritable ulcer, there is a deficiency of secretion of the solid material of reparation, and a tendency to excess of organization. The granulations are small and florid, bleed readily, and are very sensitive to the touch; in the other form, the ulceration partakes of the phagedænic character. Mr. Skey regards "every form of ulcer not caused by external injury, that is, interrupted in its healing by causes not external, as, in one sense, constitutional; that is to say, they take the type of the general health, and are remediable by internal therapeutic or dietetic agency." He does not include those forms of ulcer which may be justly termed *specific*.

In reference to the *treatment*, which forms the subject of the next chapter, Mr. Skey says, "although I conceive that it will be found applicable to every form of ulcer dependent upon weakness,—and in this catalogue I should include more than nine-tenths of those affecting the lower limbs, and a large proportion of ulcers situated on any other part of the body,—yet there is none in which it will be found so striking as in that most frequent form of the chronic or callous ulcer affecting the legs of old persons."

We cannot pass entirely without notice the remarks which, in this part of his essay, the author has made on Mr. Baynton's long-tried and very valuable treatment of certain forms of ulcers. He believes that Mr. Baynton had no very distinct notion of the principle of the application of his treatment; and says, that the moderate and uniform pressure recommended does not produce its good effect "*by supporting the granulations* by stimulating the functions of the skin," because healthy granulations require no support: however plausible the idea, there is nothing in the structure of a healthy granulation that demands the assistance of art, to the end of rendering it efficient for the purposes by which it is designed by nature. The effect of pressure applied to the surface of an ulcer is to excite to action, to rouse, to stimulate." The author appears to forget that the question is not, what healthy granulations demand, but what unhealthy granulations require. We are not compelled to believe that pressure is a stimulant, because unhealthy granulations assume an improved appearance under its use; but we know that the action of the heart, in propelling the blood from within outwards towards the skin, acts with a uniform pressure upon its interior surface. This pressure is, of course, resisted by the skin when entire, and the blood flows in its proper channels; but, when an aperture is made, there is no longer any resistance at that point, and consequently the blood flows out of it from all points of its circumference, because the internal pressure, being uniform, cannot act upon any particular point. Now, as in the case of an ulcer, the assistance afforded by the skin, which supports the vessels, is wanting, we can readily understand that the uniform and moderate support afforded by the adhesive plaster, as employed by Mr. Baynton, does actually in this respect simulate the function of the skin, and compensate for its absence as far as can be done by artificial means; and also that the plaster, in thus supporting the vessels, may have a distinct tendency to relieve inflammation by restoring the blood to its proper channels, and consequently to improve the appearance of granulations.

The second division of the essay is devoted to a consideration of some of the medical properties of Opium. Its acknowledged efficacy in senile gangrene, as first recommended by Pott, led Mr. Skey to reflect on its applicability to ulcers. In small doses, it promotes genial warmth, and, by giving energy to the extreme arteries, maintains an equable balance throughout every part of the body. Its beneficial influence on the physical frame requires the previous existence of actual disease, the ravages of which demand assistance from the powers of renovation. But (and the author particularly and most judiciously insists on this point,) the effects above described are only obtained by the most moderate dose. In this dose, its powers as a stimulant are followed by no perceptible depression, either of the pulse or nervous system; and in such quantity only does the author recommend its use. The power possessed by it, when thus employed, he speaks of as one "rousing the dormant energies of local health through the means of the circulating system." To those who are disposed to contend that the influence of opium is more exercised upon certain organs, Mr. Skey replies,

"I deny the influence of opium upon either the brain, genital organs, or skin, when administered in doses of half a grain, night and morning, to adult subjects, with open and discharging wounds. These patients are ordinarily most tolerant of its influence, and, in the doses in which I recommend its use, are insusceptible of such eccentric action. I have been convinced of the truth of this assertion by repeated failure in the treatment by opium of cases of irritable and not ulcered legs, with dryness and desquamation of the cuticle. In these cases, I have frequently known the malady aggravated rather than relieved by opium. The dose should hold a relation to the state of the wound and of the constitution, and should always be an absolutely small one, although it may be comparatively large."

The former part of the above extract, which contains but the statement of what has fallen under the author's own observation, is well worthy of attention, and probably may be of very extensive application; but the latter, in which he justifies his conviction of its truth, is an instance—unfortunately too frequent in medical writings,—of the illogical inferences which may be derived from careless analogies. Opium, says Mr. Skey, may be continued with advantage, whilst the constitution requires artificial power. So long as there exists a drain on the circulation, the influence of opium on the general health will be found most beneficial. But he would wish that the conditions should be well defined in which its beneficial effects are or are not to be expected. "It is less efficient in florid or sanguineous temperaments; not that its influence is less marked, but because such habits are usually deranged by all narcotics. It is less efficient in youthful age or childhood, and it is objectionable in cases in which malignant disease is present in the system. Excluding these exceptions, all of which are subject to other exceptions, the wards of the metropolitan hospitals teem with cases of ulceration amenable to the treatment by opium, in which, perhaps, the disease is situated in the lower extremities." Of these, such a case as the following is described: that of a man beyond the middle of life, his nervous system exhausted, his leg or legs exhibiting, to any extent between the knee and the ankle, a dark red-brown discoloration of the skin, surrounding a deep excavated ulcer, immediately encircled by whitish elevated integument; the base of the ulcer being flattened or ragged, covered by a layer of watery lymph, and secreting a various quantity of fluid. There are no granulations, but an unhealthy and unorganized flake instead of them. Instead of Mr. Baynton's treatment, Mr. Skey recommends the internal, constitutional use of opium. He goes on to say that, "in many cases, a very palpable effect is produced by eight drops of the tincture twice a day; but I rarely commence with a less dose than half a grain of the extract, night and morning. Age, sex, constitution, and idiosyncrasy, of course, require attention. But in every case, however favorable, I commence with this dose. On these conditions, it may be rapidly increased, if necessary, up to two grains night and morning." Opium thus given does not constipate. As a general rule in trying the effect of this remedy, all local treatment has been suspended, excepting the application of poultices or of some unstimulating ointment. It will be found most effective in cases of chronic ulcers, in old persons inclined by habit to spirituous drinks, and the leuco-phlegmatic who have not been similarly addicted. As it regards time, its effects have been least strikingly exhibited on the legs, and most so on the head and trunk.

Mr. Skey likewise informs us that he has extended the use of opium, in the form of the tincture, in doses of from ten to fifteen drops or more, with advantage in cases of common catarrh; and he also mentions a case of what he calls passive hyperemia of the nose, "which had existed for two years in a lady, aged fifty, of languid circulation and cold extremities, and of remarkably abstemious habits," which was successfully treated by opium. We are not able to speak, from our own experience, of the employment of opium in such cases, but we do not doubt its utility in many instances where the constitution is weak and the circulation languid. Its effect, our author tells us, is to arrest the troublesome defluxion from the nose which characterizes catarrh, and that "no reaction follows this treatment. The discharge does not recur with increased violence, and, if it return at all, is considerably lessened in quantity."—"The sensible effects of the medicine," he says, "are a general glow of warmth throughout the body, with a uniform degree of perspiration proportionate to the quantity taken. That the warmth of the skin is an indication of healthy action may be inferred from this fact, that it relieves itself, if in excess, by cutaneous transpiration."

Mr. Skey has communicated a few of the cases of local disease treated by the method above recommended. Of these, nine are cases of chronic ulceration of the legs, in three of which the veins were varicose; three of open bubo; two of phagedænic ulceration. In all these cases the sores assumed a healthy character immediately after commencing the use of opium, as already recommended. The two remaining cases are mentioned to shew the efficacy of the medicine in arresting ptyalism.

As we ourselves have often derived advantage from the employment of opium in such cases, we are not disposed to examine very carefully what amount of benefit was derived, by constitutions exhausted by bodily fatigue and excess, in some of these cases, from rest, cleanliness, porter, and meat diet. Setting aside the effects of these remedies, there is evidence enough of the advantage obtained by the use of opium; and Mr. Skey deserves the thanks of the profession for having published his experience in its employment. The effects which all surgeons have frequently witnessed from the use of the drug in many forms of ulceration will dispose them to believe in its more extensive applicability, and to give it a fuller trial, according to the suggestions of Mr. Skey.

Having spoken favorably of this essay in a practical point of view, we can find nothing else to commend. What little pathology there is in it is sadly unpathological; much of its reasoning is very illogical; and, generally speaking, its composition is so defective and so little indicative of a habit of clear and connected thought, that we have been obliged to read again and again in some instances, still remaining in doubt as to whether we have always correctly appreciated its author's meaning.

---

